# Density‐dependent demography and movements in a cyclic brown lemming population

**DOI:** 10.1002/ece3.9055

**Published:** 2022-07-04

**Authors:** Dominique Fauteux, Gilles Gauthier

**Affiliations:** ^1^ Canadian Museum of Nature Ottawa Ontario Canada; ^2^ Centre d'Études Nordiques and Université Laval Québec Québec Canada

**Keywords:** food web, rodents, small mammals, top‐dhown regulation, trough phase, tundra

## Abstract

Theoretical modeling predicts that both direct and delayed density‐dependence are key factors to generate population cycles. Deciphering density‐dependent processes that lead to variable population growth characterizing different phases of the cycles remains challenging. This is particularly the case for the period of prolonged low densities, which is inherently data deficient. However, demographic analyses based on long‐term capture–mark–recapture datasets can help resolve this question. We relied on a 16‐year (2004–2019) live‐trapping program to analyze the summer demography and movements of a cyclic brown lemming population in the Canadian Arctic. More specifically, we examined if inversely density‐dependent processes could explain why population growth can remain low during the prolonged low phase. We found that the proportion of females in the population was inversely density‐dependent with a strong male‐biased sex ratio at low densities but not at high densities. However, survival of adult females was higher than adult males, but both had lower survival at low densities than at high ones. Distances moved by both adult males and females were density‐dependent, and proportion of females in reproductive condition was weakly density‐dependent as it tended to increase at low density. Individual body condition, measured as monthly change in body mass, was not density‐dependent. Overall, the strong male‐biased sex ratio at very low densities suggests a loss of reproductive potential due to the rarity of females and appears to be the most susceptible demographic factor that could contribute to the prolonged low phase in cyclic brown lemmings. What leads to this sex‐bias in the first place is still unclear, potentially owing to our trapping period limited to the summer, but we suggest that it could be due to high predation rate on breeding females in winter.

## Graphical abstract

Density‐dependence is often difficult to measure during the low abundance phases of fluctuating populations, but we present new evidence that inverse density‐dependent sex‐ratios ‐ i.e. proportion of adult females in the population increasing with density ‐ characterises brown lemming cycles. Such low abundance of females compared to males remain unexplained, but may have strong implications in preventing a low density population to grow again under delayed density‐dependant predation.

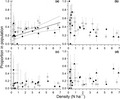

## 1 INTRODUCTION

Theory predicts that population cycles observed in herbivore populations are driven by density‐dependent processes that are either caused by extrinsic or intrinsic factors (Stenseth, 1999). Among the different phases of population cycles, the most obscure and enigmatic one remains the prolonged low abundance phase that can last for several years after the decline in some species (Barraquand et al., 2017; Boonstra et al., 1998). From theoretical models, one can explain the low phase by delayed density‐dependent effects of factors such as predation or low food abundance (Bjornstad et al., 1995; Sheriff et al., 2009; Stenseth, 1999) and phase‐dependent factors (Barraquand et al., 2014), such as delayed maturation (Ergon et al., 2001; Ergon et al., 2011). However, empirical evidence in support of changing density‐dependence across phases is still lacking especially because of the difficulty to study populations at their lowest densities. Some support for the specialist predator hypothesis through a delayed response was found to explain the low phase in cyclic lemmings and voles (Gilg et al., 2003; Norrdahl & Korpimäki, 2000), but others have rejected this hypothesis (Graham & Lambin, 2002; Mougeot et al., 2019). Non‐lethal effects, such as reproduction impairment that may last over several generations through maternal effects, have gained support, especially for snowshoe hare (*Lepus americanus*, Sheriff et al., 2009) and more recently for meadow voles (*Microtus pennsylvanicus*; Edwards et al., 2021; but see Boonstra & Boag, 1992; Fauteux, Gauthier, Berteaux, et al., 2018). The current lack of empirical evidence for changes in demography and population structure in cyclic species at low versus high densities is hampering our understanding of why these populations are susceptible to prolonged low phases.

Detailed demographic analyses of populations in relation to densities are useful to disentangle what factors are responsible for population growth in cyclic species (Aars & Ims, 2002; Fauteux et al., 2015; Hodges et al., 1999). By identifying how survival, dispersal, and reproduction are changing with density, we can infer the most plausible cause of slow growth at low density based on known relationships between specific demographic traits and various extrinsic (e.g., predation) and intrinsic factors (e.g., social interactions). Here, we address this question using a live‐trapping dataset collected on brown lemmings (*Lemmus trimucronatus*) in the Canadian Arctic over 16 years (2004–2019), the longest capture–mark–recapture time series of lemmings in the Arctic. In this population, brown lemmings show regular, high amplitude cycles of abundance with a 3–4 years periodicity (Gauthier et al., 2013). Our objectives were to determine if summer demographic parameters or movement are density‐dependent, and if so, which one could contribute to slow population growth at low densities. In other words, we were particularly interested in identifying inversely density‐dependent parameters during the summer period.

Based on past studies highlighting the importance of predation in causing the decline phase of cyclic lemmings and northern voles (Fauteux et al., 2016; Gilg et al., 2003; Norrdahl & Korpimäki, 1995; Wilson et al., 1999), we hypothesized that the lack of population growth at low density could be caused by both direct effects of generalist predators (e.g., Arctic fox, *Vulpes lagopus*) and the delayed density‐dependent response of mustelids (Hanski et al., 1991). Under this hypothesis, we expected an inversely density‐dependent survival (i.e., lowest survival at low density) in lemmings caused by the delayed numerical and functional responses of mustelids (Gilg et al., 2006).

Animal movement can be an important factor affecting vulnerability to predation. Adult males are known to be more active and more mobile than females (Banks et al., 1975), which can lead to increased exposure to predation as seen in voles (Norrdahl & Korpimäki, 1998). In addition, when densities reach extremely low levels (<0.1 lemming/ha), lemmings may rarely encounter conspecifics within their usual home range (0.5–1.5 ha; Banks et al., 1975), which may force them to move or disperse over longer distances to find mates (Andreassen & Ims, 2001; Ostfeld & Canham, 1995). Consequently, we expected that (1) males should move more than females, (2) all lemmings should move more at low densities, (3) males should have lower survival than females, especially at low densities, and (4) this should lead to a female‐biased sex‐ratio at low density.

Aside from predation, other factors have been proposed to be responsible for the low phase of cycles such as lack of food following overgrazing at high density, maternal effects, or parasites, though evidence of delayed density‐dependent effects of parasitism was observed in Lepidopterans and not rodents (Klemola et al., 2014). These factors could also lead to lower survival at low densities than at high ones, but, in contrast to the predation hypothesis, these factors should also lead to less healthy animals (i.e., lower body condition or growth), and possibly lower reproduction at low density. If one of these hypotheses were correct, we thus expected that body mass gain and proportions of females in reproductive condition during the summer should be lower at low than at high densities.

## 2 MATERIAL AND METHODS

### 2.1 Study system

Our study was conducted in the Qarlikturvik Valley on Bylot Island, Nunavut, Canada (73°08′N, 80°00′W). Only two rodent species are present: brown lemmings, which are mostly found in wet and mesic tundra areas, and collared lemmings (*Dicrostonyx groenlandicus*) that are mostly found in the mesic habitat and drier hills. Both species are cyclic (Gauthier et al., 2013), but the brown lemming has much larger population fluctuations, increasing by up to 100‐fold between low and high densities, and is the most abundant of the two species. Maximum densities of brown lemmings may reach up to ~15 ha^−1^ in peak years while collared lemmings may reach 1 ha^−1^. Although winter data are scarce, previous studies showed that growth phases typically occur in winter, while decline phases most likely occur in late summer and Fall (Fauteux et al., 2015). Competition between both species favors brown lemmings (Morris et al., 2000). For those reasons, we focused our study only on brown lemmings. Their main predators are Arctic foxes, ermines (*Mustela erminea*), snowy owls (*Bubo scandiacus*), long‐tailed jaegers (*Stercorarius longicaudus*), and rough‐legged hawks (*Buteo lagopus*). On Bylot Island, brown lemmings feed mainly on willows (e.g., *Salix arctica*), mosses (e.g., *Aulacomnium* sp., *Polytrichum* sp.) and, to a lesser extent, grasses (e.g., *Alopecurus* sp., *Arctagrostis* sp.; Fauteux et al., 2017; Soininen et al., 2015).

### 2.2 Lemming live‐trapping

From 2004 to 2019, lemmings were live‐trapped from June to August in two 11‐ha trapping grids, one located in wet tundra and the other in mesic tundra (also called mesic grid 1). Each trapping grid consisted of 144 trapping stations spaced out every 30‐m according to a Cartesian plane (12 × 12) and each station had one Longworth live‐trap. Starting in 2007, a third trapping grid made of 96 trapping stations (8 × 12) was added in the mesic tundra habitat (also called mesic grid 2). In 2013–2019, this 9‐ha grid was fenced and covered by a net made of fishing lines to prevent predators (birds of prey, foxes) from accessing the lemmings (hereafter the predator exclosure), creating specific conditions for this grid in those years (Fauteux et al., 2016). However, the exclosure was permeable to ermines as it was designed to allow dispersal by lemmings. Thus, we added a fourth level to the trapping grid covariate in the analyses corresponding to years with a predator‐exclosure. Capture–mark–recapture schedules consisted of three primary periods (four in the first 4 years) and up to 10 secondary periods (i.e., traps being visited every 12 h) per primary period each summer. All lemmings captured were identified, sexed, weighed, aged, their reproductive condition noted and marked with a passive integrated transponder or an ear‐tag. More details on live‐trapping schedules, baiting, and marking lemmings can be found in Appendix S1. All field manipulations and animal care precautions were approved by the Animal Welfare Committees of Université Laval and the Canadian Museum of Nature, and by Parks Canada.

### 2.3 Densities and sex and age ratios

We estimated densities of adult and juvenile males and females with spatially explicit capture–recapture (SECR) models for each primary period, grid, and year (Efford, 2004). In the models, we used a 100‐m buffer that corresponds to three to four times the daily movement of lemmings and a half‐normal detection function. For the high abundance years, densities were estimated with separate SECR models for each age and sex category and trapping grid. For the low abundance years, which typically have ≤5 individuals captured per primary period, we combined datasets from all age and sex categories for each trapping grid due to low sample size and assumed that the probability of capture and the movement parameter (sigma) were constant among lemmings, but different among trapping grids. To further reduce the number of parameters, densities were derived from models that used the conditional likelihood (Borchers & Efford, 2008).

We calculated the proportion of each sex and age category from the densities obtained with the SECR models and estimated their variance by bootstrapping. We repeated the SECR analyses 200 times with a different randomized dataset each time. Each dataset was obtained by resampling, with replacement, individual capture histories (i.e., histories were not changed) of the original datasets while keeping the same sample size. The bootstrapping was repeated for each primary period, grid, and year. We calculated the proportions of each sex and age group from the resulting densities, and the mean and variance of these proportions across the 200 iterations, from which we derived the standard errors.

### 2.4 Distances moved

For each individual, we calculated the maximum and average linear distance between the point of first capture and all subsequent recaptures on each trapping grid. When individuals were captured in more than one primary period of the same year, captures from all primary periods of that year were pooled. Ultimately, we obtained one maximum and one average distance moved per individual. Trapping grids were separated by >600 m and no lemming was ever captured in more than one trapping grid.

### 2.5 Reproduction

We analyzed the reproductive condition of adult females only because the condition of males was not noted systematically during all sampling years and because of the importance of females to population dynamics in general. From 2009 to 2019, captured females were classified as non‐reproductive (no sign of past or current reproduction) or reproductive (perforate vagina, lactating or showing enlarged nipples, or pregnant with an enlarged abdomen and palpable fetuses).

### 2.6 Daily change in body mass

We measured the daily change in body mass (g) of lemmings between consecutive periods from the difference between the body mass at the primary trapping period *t* + 1 and the body mass at the primary period *t* divided by 20 or 30 days, depending on the time between primary periods. Individuals recaptured but in non‐consecutive primary periods (e.g., captured in June, not captured in July, recaptured in August), were ignored for this analysis. If an individual was captured more than once within a primary period, we averaged its body mass. Because primary trapping periods were separated by either 20 or 30 days with traps locked open without any bait added during the interval, we assumed that trap‐related effects on body mass were negligible. Pregnant females were excluded from the analysis.

### 2.7 Statistical analyses

We modeled the influence of total population density on the proportion of each age/sex group with a robust linear model where extreme values were given a weight based on residuals with the *M*‐estimator (Huber, 1981; Venables & Ripley, 2002). The trapping grid was added as a covariate. To consider the errors of the data points on both axes, we used a bootstrapping approach to obtain the regression coefficients and their 95% confidence intervals. We first generated 2000 new datasets, each with the same sample size as the original, by resampling with replacement paired population density and age/sex proportion values in our datasets. Because each of these paired densities and proportions had errors, we further propagated the error by replacing them with a random value obtained from a normal distribution generated with the observed densities and proportions and their respective standard errors. This randomization was performed on the logit scale for proportions and on the log scale for densities prior to back‐transforming values to the real scale. We applied the robust linear model on each of the randomly generated datasets and estimated regression coefficients as the mean from the 2000 models and their 95% confidence interval boundaries as the 2.5% and 97.5% quantiles (i.e., 50th and 1950th predicted values in ascending order). The package “*MASS*” in the software R was used to run the robust linear models (Venables & Ripley, 2002). All the following analyses were run in the R software as well, except for survival estimations.

We used the software E‐Surge (Choquet et al., 2009) to estimate summer survival probabilities among primary periods. Overwinter survival could not be estimated due to extremely low recapture rates between summers (<1%). We elaborated a set of candidate models to test the effects of sex, age, trapping grid, year, and primary period on survival. For this analysis and the following ones, the selected model was the simplest (i.e., least number of parameters) among the most parsimonious models (ΔAICc < 2) to avoid retaining uninformative parameters (Arnold, 2010). We used unequal time intervals to consider that primary periods were separated by 20 days from 2004 to 2007 and 30 days afterward. We could not directly test the relationship between monthly survival and SECR population density due to our complex dataset and the definition of the design matrices in E‐Surge. Instead, we conducted a posteriori analysis using a robust linear model relating survival probabilities estimated between primary periods *t* and *t* + 1 for each year, and sex‐age groups with densities at *t*.

We analyzed the maximum and average distance moved during the summer between the first capture of individuals and their subsequent recaptures. When all captures were at the same trap, a value of 0 was assigned to that individual. Due to the many zeros inherent to such data, we used a negative binomial regression. A set of candidate models was developed with additive and interactive effects of sex, age, trapping grid, and annual population density (i.e., average of July and August densities). We controlled for unequal number of recaptures between individuals with an offset (log‐transformed total number of captures). Model selection was conducted in the same way as for the survival analysis.

For each adult female that was reproductive when captured, a value of 1 was attributed, and a value of 0 when non‐reproductive. We used a mixed‐effects binomial model with individuals as the random variable to consider the repeated measures taken on them. The set of candidate models included additive effects of population density, primary period, and trapping grid as fixed effects to control for when and where lemmings were captured. The top model was selected using the same approach as for the other analyses.

We tested whether change in body mass was density‐dependent with linear mixed‐effects models where individuals were also used as the random variable. All candidate models included the initial body mass of lemmings as a fixed variable to consider the more rapid growth of young individuals compared to adults. The other fixed variables included additive or interactive effects of sex, primary period, trapping grid, and population density to consider ontological, seasonal, spatial, and density‐dependent effects. Model selection was conducted in the same way as for the previous analyses. All model coefficients and estimates are reported with their 95% confidence intervals in brackets.

## 3 RESULTS

### 3.1 Sex and age ratio

**FIGURE 1:**
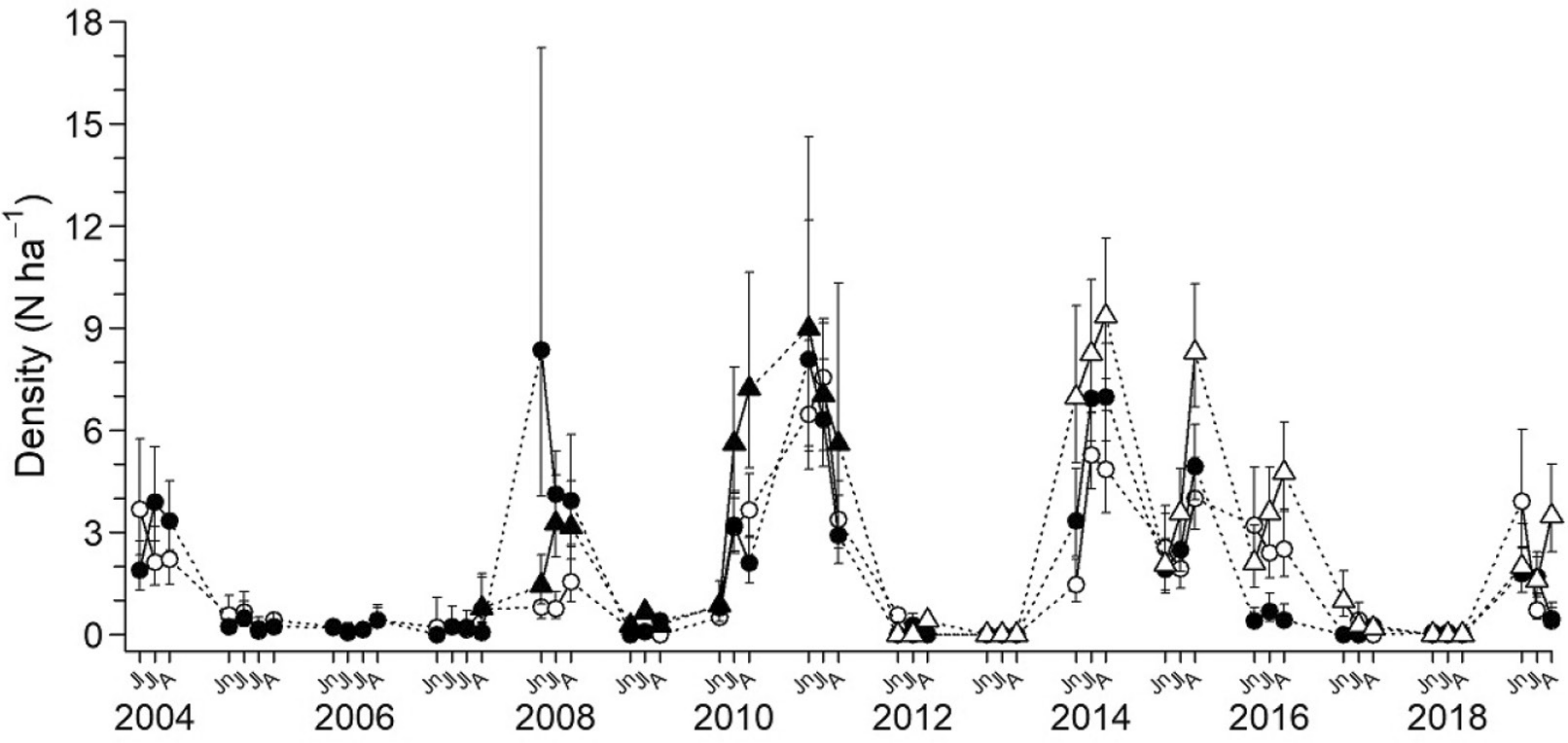
Brown lemming densities at Bylot Island over time with their 95% confidence intervals in the wet meadow trapping grid (black circles), Mesic trapping grid 1 (white circles), Mesic trapping grid 2 (black triangle), and predator exclosure trapping grid (white triangles). Densities were estimated with spatially explicit capture–recapture models. Jn = June, Jl = July, A = August. In 2004–2007, live‐trapping and density estimations were conducted for early July late July, and mid

**FIGURE 2:**
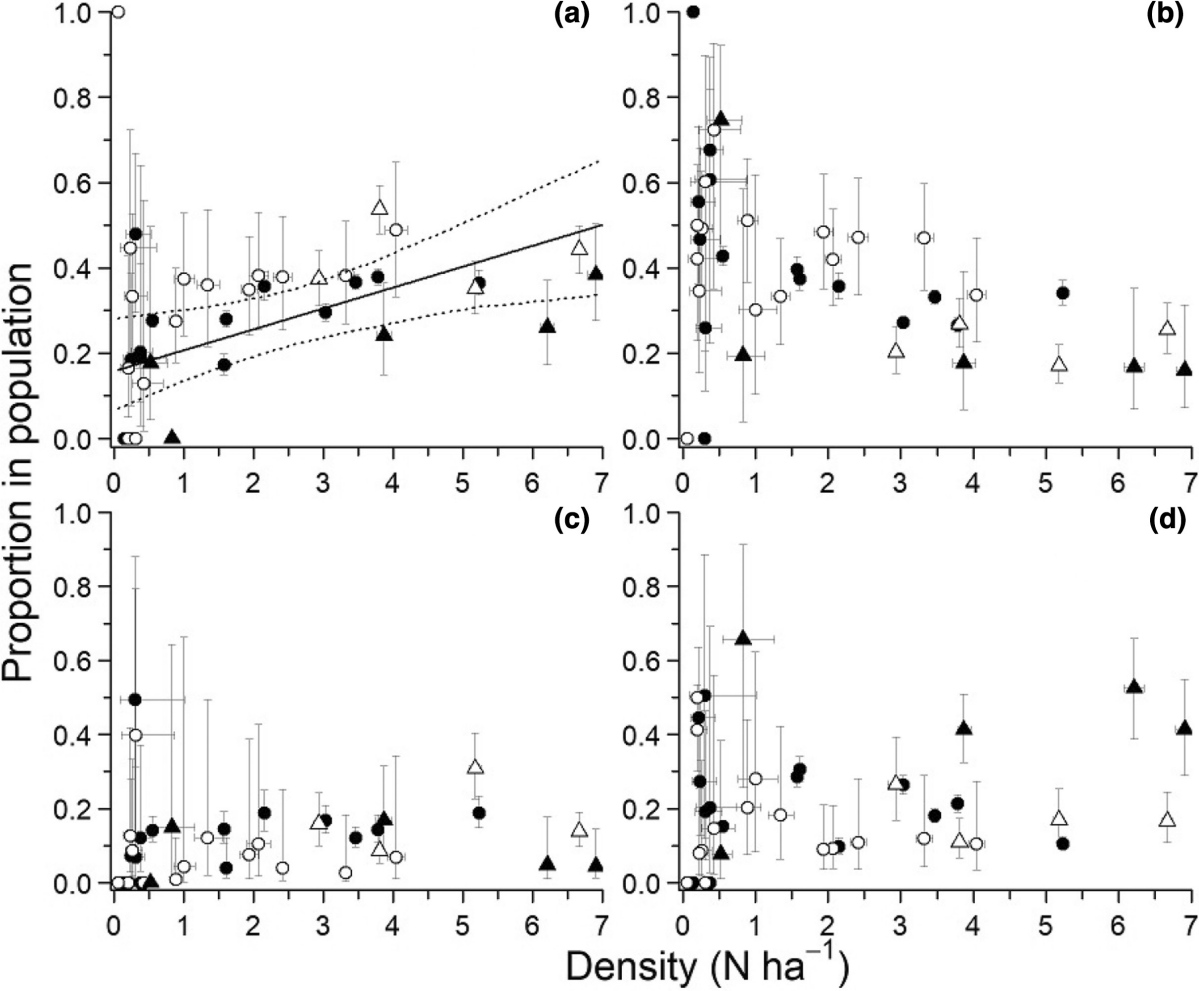
Proportion of adult females (a; ≥28 g), adult males (b; ≥30 g), juvenile females (c), and juvenile males (d) in the population in relation to total population density (i.e., sum of densities of all lemming sex and age classes) at Bylot Island, 2004–2019. Solid lines represent the robust regression estimated by bootstrapping and dotted lines represent the 95% confidence intervals (absence of lines indicates non‐significance). Black circles: Wet meadow trapping grid; white circles: Mesic trapping grid 1; black triangles: Mesic trapping grid 2; white triangles: Predator exclosure trapping grid

Densities of brown lemmings on the different grids varied throughout the years, going from local extirpation in 2013 to a maximum of 9 lemmings ha^−1^ in 2014 (Figure 1). Sample sizes are presented in the Appendix, Table S1. The proportion of adult females in the population was positively related to population density (*β* = 0.049, [0.014, 0.078]; Figure 2). In contrast, proportions of adult males (*β* = −0.034, [−0.080, 0.006]), juvenile males (*β* = −0.009, [−0.055, 0.030]), and juvenile females (*β* = 0.011, [−0.015, 0.034]) did not vary significantly with density. At high densities, the female: male ratio of adults was close to 1:1, but it was approximately 1:3 at low densities. The age ratio was generally in favor of adults with, on average, 2.5 adults per juvenile (Appendix S1, Figure S1).

### 3.2 Survival

**FIGURE 3:**
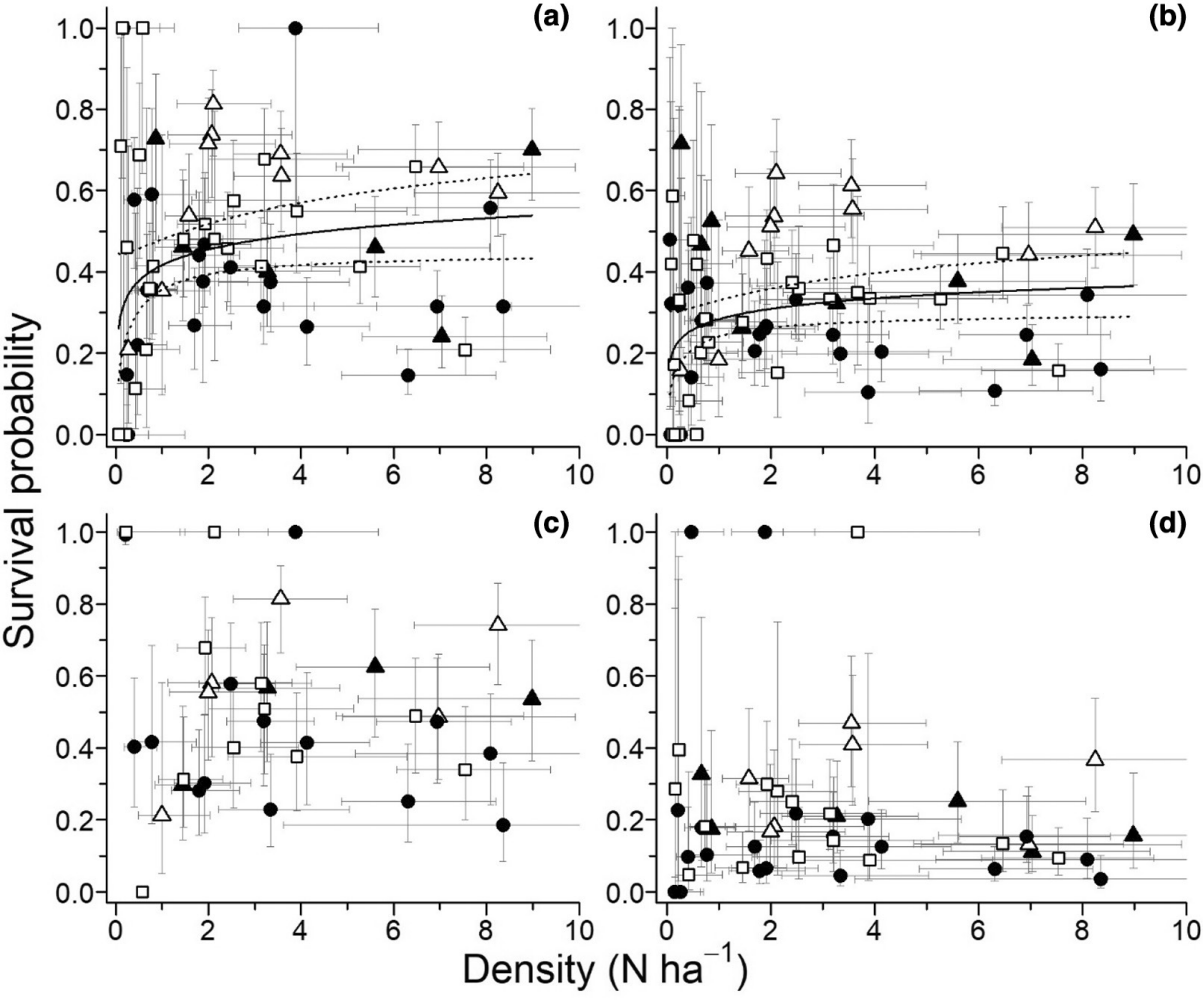
Monthly summer survival probabilities of adult female (a), adult male (b), juvenile female (c), and juvenile male (d) brown lemmings in relation to population density at Bylot Island, 2004–2019. Solid lines represent a significant relationship from the robust regressions and dotted lines represent the 95% confidence intervals (absence of lines indicates non‐significance). Black circles: Wet meadow trapping grid 1; white circles: Mesic trapping grid 1; black triangles: Mesic trapping grid 2; white triangles: Predator exclosure trapping grid. Gray lines are the 95% confidence intervals on both axes for each observation

The most parsimonious model from the survival analyses included full‐time effects (i.e., variations among each month and year), an interaction between lemming age‐sex groups and primary periods, and an additive effect of trapping grids (Appendix, Table S2, Figure S2). Adult females had higher apparent monthly survival ($\hat{s}$ = 0.46, [0.43, 0.48]) than adult males ($\hat{s}$ = 0.29, [0.27, 0.32]), whereas the converse was found in juveniles (females, $\hat{s}$ = 0.50, [0.47, 0.53]; males, $\hat{s}$ = 0.22, [0.19, 0.25]). Monthly survival of adults was slightly lower in late ($\hat{s}$ = 0.30, [0.25, 0.35]) summer compared to early summer ($\hat{s}$ = 0.39, [0.27, 0.52]), whereas the opposite was true for juveniles ($\hat{s}$ = 0.42, [0.38, 0.45] vs. $\hat{s}$ = 0.26, [0.10, 0.52]). Apparent monthly survival was highest in the predator exclosure grid ($\hat{s}$ = 0.48, [0.45, 0.52]) and lowest in the wet grid ($\hat{s}$ = 0.29, [0.27, 0.31]). Capture probability was estimated at 1.00 ([1.00, 1.00]) for males and 0.88 ([0.78:0.93]) for females. The a posteriori analysis of the relationship between apparent monthly survival and density shows that survival increased with density in both adult females (*β* = 0.056, [0.007, 0.104]) and males (*β* = 0.037, [0.008, 0.066]; Figure 3). It is noteworthy that all lemmings captured in 2012 and 2018 (*n* = 17), 2 years of very low density, were never recaptured between primary periods, leaving those years to be the only ones with a survival probability of 0. Survival of juvenile females (*β* = −0.028, [0.110, 0.054]) and males (*β* = −0.002, [−0.030, 0.033]) were not related to density.

### 3.3 Movements within trapping grids

**FIGURE 4:**
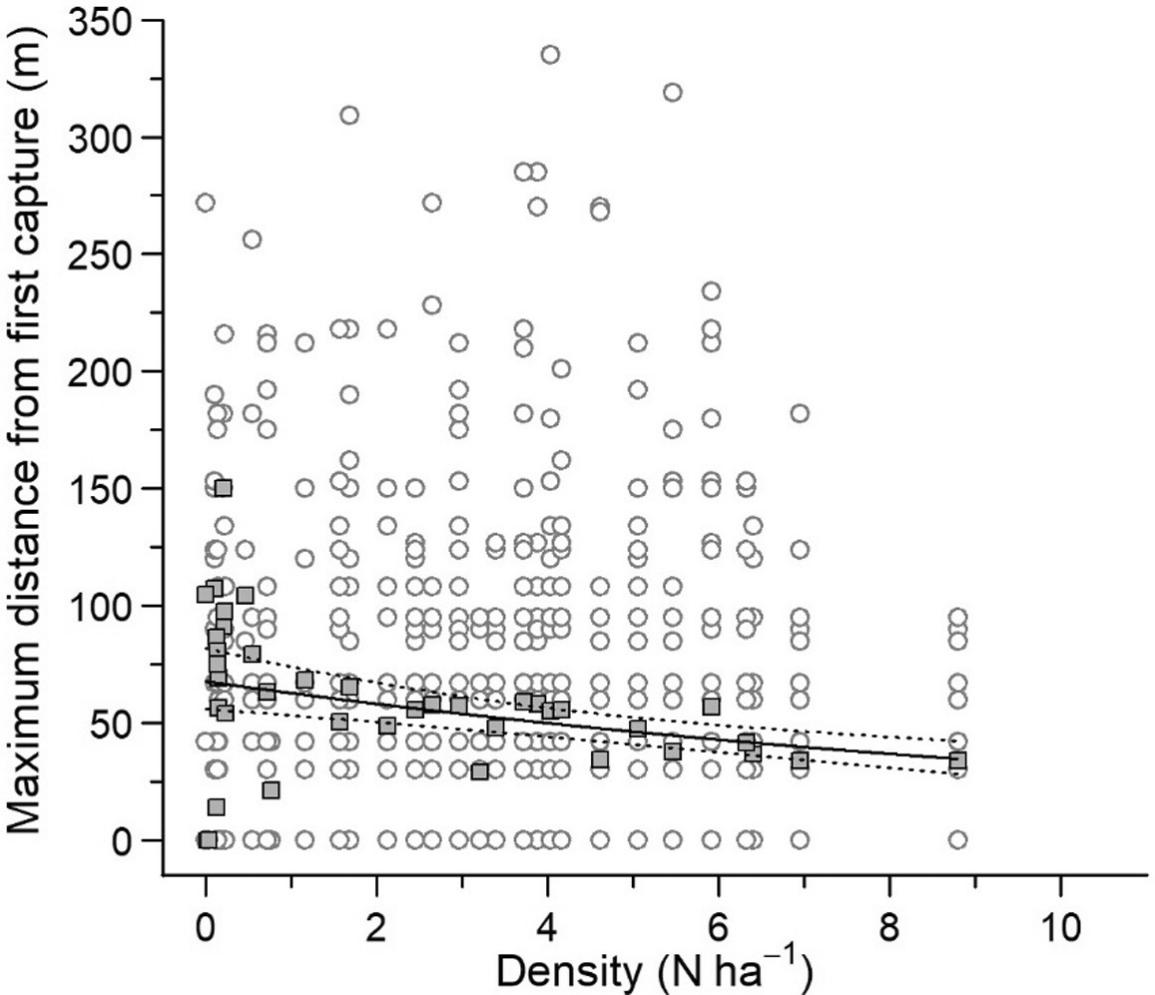
Maximum distance (m) between the initial capture and any recapture of brown lemmings during the same summer in relation to annual population density at Bylot Island, 2004–2019. For better visualization of the raw data, empty gray circles represent the maximum distance of each individual and filled gray squares are the averaged maximum distance across all individuals for each year. The solid black line represents the negative binomial regression and dotted lines represent the 95% confidence intervals

The most parsimonious model of the analysis of maximum distance moved included a negative effect of density (*β* = −0.076, [−0.112, −0.040]) and an interaction between sex and age (*β* = −0.497, [−0.968, −0.039]; Figure 4; Appendix S1, Table S3). Maximum distances moved by lemmings decreased from 78 ± 63 m (standard deviation) at very low density to 48 ± 49 m at high density. Maximum distance moved was highest in adult males (66 ± 56 m), intermediate in adult females (41 ± 42 m), and lowest in juveniles (males: 25 ± 32 m, females: 22 ± 36 m). Similar results were obtained with average distances (density: *β* = −0.068, [−0.105, −0.031]; interaction between sex and age: *β* = −0.62, [−1.111, −0.152]).

### 3.4 Reproductive conditions

**FIGURE 5:**
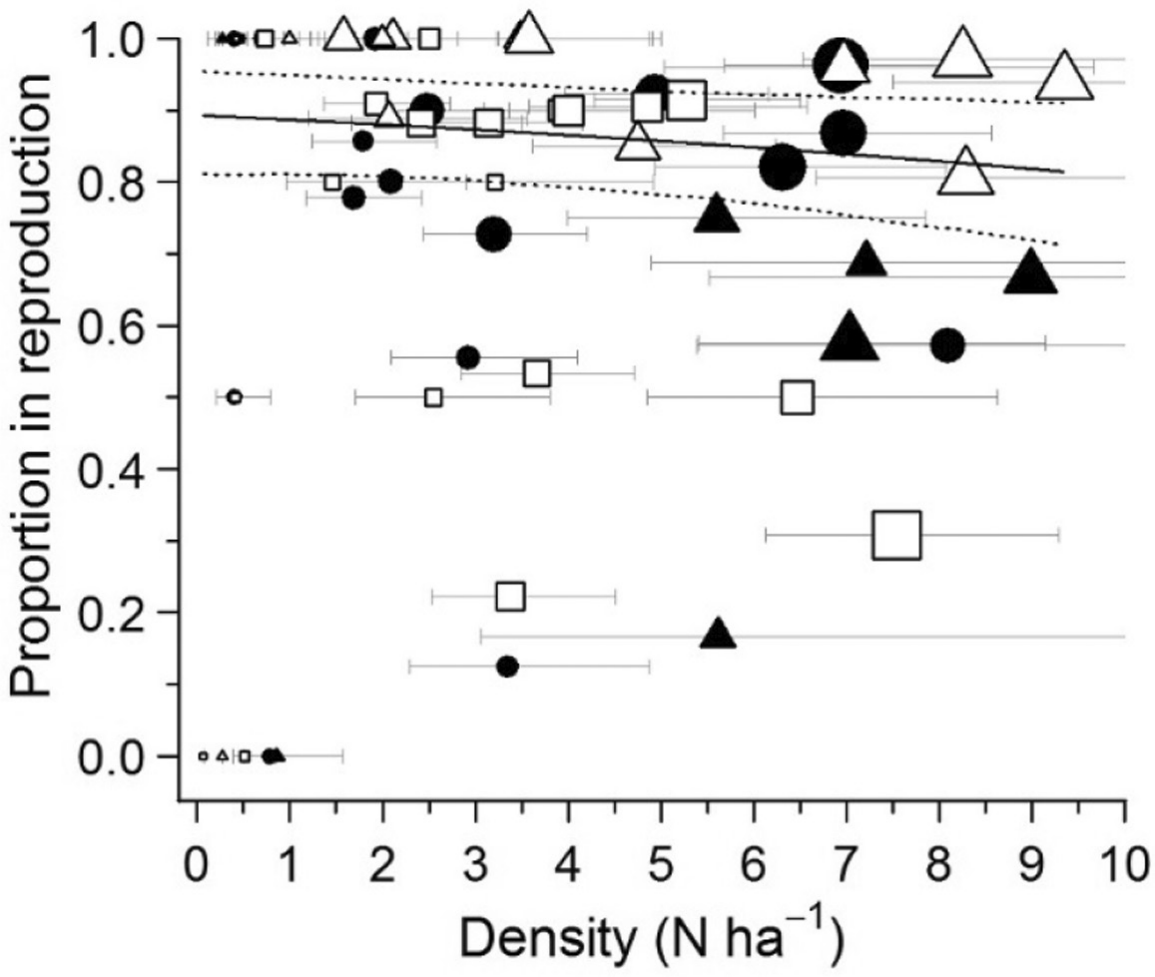
Proportion of adult females in reproductive condition (i.e., with perforate vagina, lactating, or pregnant) per trapping grid, primary period, and year in relation to the total population density at Bylot Island, 2009–2019. The solid line represents the predicted values from the binomial model and the dotted lines are the 95% confidence intervals. Black circles: Wet meadow trapping grid 1; white squares: Mesic trapping grid 1; black triangles: Mesic trapping grid 2; white triangles: Predator exclosure trapping grid. Size of points represent sample size with the smallest being 1 to the largest being 54

The most parsimonious model for the proportion of adult females in reproductive condition analysis included the variables density, primary periods, and trapping grids (Appendix, Table S4). The proportion of adult females in reproductive condition slightly decreased with density (*β* = −0.090, [−0.170, −0.004]; Figure 5; Appendix, Table S5). The proportion of reproductive females in mid‐July was higher than in mid‐June but not compared to mid‐August. Finally, the proportion of reproductive females was lower in the mesic trapping grid (0.67) than in the wet trapping grid (0.81) and was highest in the predator exclosure (0.94).

### 3.5 Daily change in body mass

**FIGURE 6:**
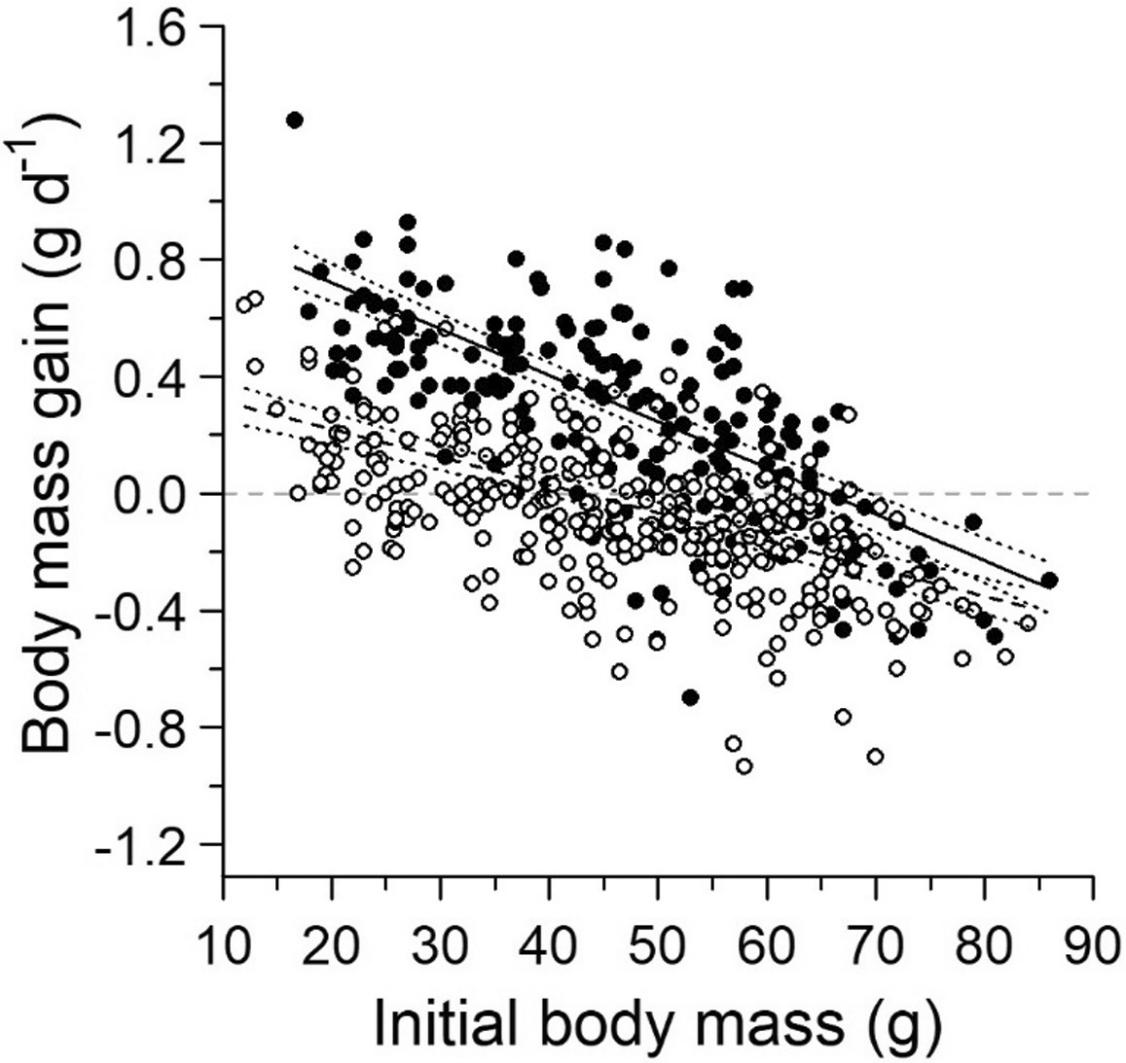
Predicted (slopes) and observed (circles) daily gain in body mass of lemmings in relation to initial body mass for early summer (June–July; filled circles, solid line) and late summer (July–August; open circles, dashed line) at Bylot Island, 2004–2019. Predictions and their 95% confidence intervals (dotted lines) are based on the most parsimonious and simplest linear mixed‐effects model. The horizontal gray dashed line is presented for visual guidance only

The most parsimonious model for change in body mass (g/day) included an interaction (*β* = 0.006, 95% CI = [0.004:0.009]) between initial body mass and primary period (June–July vs. July–August), suggesting both ontological and seasonal effects, but no relationship with population density (Figure 6; Appendix, Table S6). Young (i.e., <30 g) lemmings gained less mass in late than in early summer, whereas adults generally gained mass in early summer but lost mass in late summer, especially among the largest lemmings. The change in body mass was similar between the wet tundra and the predator exclosure trapping grids but lower in the two mesic grids.

## 4 DISCUSSION

### 4.1 Puzzling summer demography

As expected, movements were density‐dependent for all lemmings, and males moved longer distances than females, supporting the hypothesis of increased efforts by males to find mates at low density. In accordance with this result, we also found that adult males had a lower survival rate than adult females during the summer. Although a posteriori analyses must be interpreted with care, there was some evidence for an inversely density‐dependent survival rate in adults, with a slightly lower survival at low density. Sex‐ratio was strongly dependent on density but, contrary to our initial prediction and the previous results, it was heavily biased in favor of males at low density. This suggests a large reduction in the number of adult females in the population compared to adult males at low densities. This shift in sex ratio is counter‐intuitive and our data did not allow us to pinpoint the precise demographic mechanism that led to it. However, considering that females contribute more to population growth than males, especially in polygynous, multivoltine species like small mammals (Erlinge et al., 2000), this reveals a reduced reproductive potential in the population at low density. A rarity of females combined with the reduced survival of adults at low population density (<1 lemming/ha) is likely to lead to a slow recruitment in the population and could be an explanation for the prolonged low abundance phase. Finally, both body mass gain and proportion of female in reproductive condition were density‐dependent, which suggests that non‐lethal factors may limit population growth at high densities but can hardly do so at low densities. We now explore potential explanations for the unexpected male‐biased sex‐ratio at low densities.

### 4.2 Trappability and mobility

We found that adult females moved over a shorter distance than males as reported in other studies on small rodents (Banks et al., 1975; Koivunen et al., 1996). A higher mobility of males may potentially lead to different detectability than females if not taken into account. However, capture probabilities of lemmings estimated in the survival analysis were high with females (0.88) having a slightly lower capture probability than males (1.0). Moreover, if low mobility reduces trappability, then the increased movements at low density observed here for all lemmings should have led to higher trappability of both males and females, not less. Thus, evidence that different trappability between sexes could explain the male‐biased sex‐ratio at low density is unconclusive.

### 4.3 Sex‐specific mortality and predation

The lower summer survival of adult males compared to females may be explained by their higher mobility, which increases their vulnerability to predation. During the summer, a large proportion of lemming mortality is due to predation by avian predators (Therrien et al., 2014). In Barrow, Alaska and in western Finland, studies on cyclic lemmings and voles have shown that males were more often hunted by avian predators, whereas females were more often hunted by weasels (Koivunen et al., 1996; MacLean et al., 1974; Norrdahl & Korpimäki, 1998; Pitelka et al., 1955). Although high avian predation could be the cause of the low summer survival of male lemmings observed here, it cannot explain the reduced adult survival at low density because many avian predators are absent during the low lemming phase (Gilg et al., 2006; Therrien et al., 2014). Considering the delayed numerical response of mustelids to small mammal population outbreaks (Gilg et al., 2003; Hanski et al., 1993), their impact should be stronger late in the cycle, namely at the beginning of the low phase, and could explain the latter result. Interestingly, recent evidence shows that even though collared lemmings are present at low densities at our study site, they represent a high proportion of items in the diet of several avian predators compared to brown lemmings, possibly due to their higher vulnerability to predation (Seyer et al., 2020). This could be indicative of apparent competition between both lemming species especially at low densities through sustained predation by ermines.

Nonetheless, it is still unclear why the sex‐ratio became highly male‐biased at low densities considering that females had higher summer survival. A possible explanation is that females may be more susceptible to predation than males at other times of the year such as in fall or winter when populations typically decline (Fauteux et al., 2015). Previous studies found that large winter nests in which females raise their young had more signs of predation by mammals (i.e., lemming bones, skin) than smaller nests with no reproduction (Bilodeau et al., 2013; Schmidt et al., 2021). Females may be more vulnerable to mammalian predators in winter due to the auditory and olfactory cues present around their nests under the snow when nursing their young (Bilodeau et al., 2013; Duchesne et al., 2011). In contrast, males may move more often and over longer distances in tunnels under the snow, which may reduce predation risks by spreading signs of activity. High predation on pregnant or nursing females could negatively affect recruitment and explain the lack of density‐dependence on proportions of juveniles at low densities even if fertility and mating success remain high (Fauteux et al., 2015; Millar, 2001).

### 4.4 Food limitation, parasites, and intrinsic factors

It is more difficult to explain the strong male‐biased sex ratio that we observed through the negative effects of starvation or infections on demography. In the High Arctic, studies revealed that lemmings have low to no impact on the vegetation they eat even during the peak abundance phase (Bilodeau et al., 2014), potentially because their maximum densities (~15 ha^−1^) never reach outbreak levels. In contrast, abundance indices of lemmings in Fennoscandia, where some evidence of overgrazing was observed after peak population years, can reach up to 30 lemmings per 100 trap‐nights, which are values much higher than maximum lemming abundance recorded at our study site (<5 lemmings per 100 trap‐nights; Fauteux, Gauthier, Mazerolle, et al., 2018; Olofsson et al., 2012; Ruffino et al., 2015). Moreover, body mass gain of both sexes did not differ between low and high density, suggesting that starvation and poor health were not more prevalent at high than at low density and vice versa. Negative physiological effects were mainly observed in small rodent populations that typically reach much higher densities (e.g., lemmings up to 200 ha^−1^ in Alaska, Pitelka & Batzli, 2007; voles up to 400 ha^−1^ in semi‐natural enclosures; Bian et al., 2015; Edwards et al., 2021). There could be sex‐specific effects of parasites mediated through endocrinal responses as observed with ticks in voles (Hughes & Randolph, 2001), but there is no evidence that such effect can have a significant impact on survival at the population level (Khokhlova et al., 2010; Steen et al., 2002). Finally, intact brown lemming carcasses were virtually never found on top of the snow in May and June, on the tundra immediately after the snow melt, or in their winter nests, suggesting minimal mortalities caused by health problems in winter.

### 4.5 Explaining the low phase

The most surprising result of our study is the presence of an inversely density‐dependent sex‐ratio strongly in favor of males at low density in cyclic brown lemmings. Pitelka and Batzli (2018) also reported for their trap samples in Alaska that male brown lemmings were in large excess compared to females during the low abundance phase, but not the high phase. Given that lemmings are polygamous and multivoltine, the high number of males and their increased movements at low density should help maintain a high mating success and prevent an Allee effect resulting from a low fertilization rate of females (Berec et al., 2007). A tendency for a higher proportion of females in reproductive condition at low density further suggests that reduced fertilization of females is not occurring at low density. We acknowledge that there may be other intrinsic factors at play that we could not measure such as fewer and smaller litters at low densities compared to high ones (e.g., Mihok & Boonstra, 1992), and such data may be increasingly accessible with new technologies such as subnivean cameras (Kalhor et al., 2021). Nonetheless, the low proportion of adult females in the population at very low density, possibly caused by higher predation on females than males in winter as argued above, must be a strong limiting factor for the reproductive potential of the population.

Interestingly, the reduced survival of lemmings at low densities contrasts with the high survival of cyclic snowshoe hares during the low phase (Hodges et al., 1999; Keith et al., 1977). A negative consequence for hares of maintaining high survival during this stressful phase is a partial suppression of reproduction (Sheriff et al., 2009). The case of brown lemmings is different in this regard because even when stressed by predators, their reproductive activity remains high (Fauteux, Gauthier, Berteaux, et al., 2018). However, the presence of ermines, a specialized predator known to show a delayed response to fluctuations in small mammal density (Gilg et al., 2003) and efficient in hunting small mammals in winter under the snow (Bilodeau et al., 2013) may be a key factor in the case of lemmings. Local extirpations that occur relatively soon after peak abundance, as observed at our study site (e.g., 2013) even if lemmings can live for up to 24 months in natural conditions (Fauteux, Gauthier, Slevan‐Tremblay, et al., 2018), is also indicative of extended periods of low survival.

The prolonged low phase of cyclic populations remains the most difficult part of the cycle to explain but comparison of our results to other studies suggests that factors involved may differ between species. However, pinpointing precisely when each phase of the cycle starts in lemmings and contrasting demographic parameters across all phases, such as between the increase and decline phases, is extremely difficult without continuous, year‐round monitoring. Although our study is limited to the summer period, it provides empirical evidence that change in population structure, and especially in sex ratio, throughout the population cycles are important parameters to consider and can provide useful clues to uncover factors driving the population dynamics. In the case of Arctic small mammals, we suggest that sex‐specific winter predation may be a key factor and should be the focus of future studies despite the challenge associated with winter field work in the Arctic. In addition to subnivean cameras, genetic analyses of lemming body remains found in winter nests, such as paws, pieces of skin, guts, or skulls, could be useful approaches.

## AUTHOR CONTRIBUTIONS

**Dominique Fauteux:** Conceptualization (lead); data curation (equal); formal analysis (lead); investigation (lead); methodology (lead); project administration (lead); resources (equal); software (lead); supervision (equal); validation (equal); visualization (equal); writing – original draft (lead); writing – review and editing (equal). **Gilles Gauthier:** Conceptualization (supporting); data curation (equal); formal analysis (supporting); funding acquisition (lead); investigation (equal); methodology (supporting); project administration (supporting); resources (supporting); software (supporting); supervision (equal); validation (equal); visualization (equal); writing – original draft (supporting); writing – review and editing (equal).

## CONFLICTS OF INTEREST

The authors declare no conflict of interest.

## CONSENT TO PARTICIPATE

No humans were involved in the study.

## CONSENT FOR PUBLICATION

No humans were involved in the study.

## CODE AVAILABILITY

All codes will be communicated on request.

## ETHICS APPROVAL

All manipulations conducted on lemmings were evaluated and approved by the Animal Welfare committees of Université Laval and the Canadian Museum of Nature, and Parks Canada.

## Supporting information


Data S1

## Data Availability

All data are currently available in open access on the NordicanaD website. Reference of the dataset: Gauthier, G. 2020. Lemming monitoring on Bylot Island, Nunavut, Canada, v. 1.3 (1994–2019). Nordicana D22, https://doi.org/10.5885/45400AW‐9891BD76704C4CE2.
