# Supplementary material for: Density‐dependent demography and movements in a cyclic brown lemming population
Source: Ecol Evol. 2022 Jul 4;12(7):e9055. doi: 10.1002/ece3.9055 (PMC9251844; doi:10.1002/ece3.9055)
Supplement: Supplementary file 1 — Data S1 [file ECE3-12-e9055-s001.docx]

**Appendix. Details on the live-trapping protocol, sample sizes, and tables and figures presenting additional results.**

*Details of the live-trapping protocol*

We used four primary periods (i.e. mid June, early July, late July, and mid August) and up to ten secondary periods (i.e. visits of traps every 12 h) in 2004-2007. In 2008, we reduced the number of primary periods to three (i.e. mid-June, mid-July, mid-August) and the secondary periods to six. Within primary periods of our live-trapping design, secondary periods consisted of visiting traps every 12 h for three to five consecutive days depending on the year. For the first primary period, traps were set in the grids for ~24 h in the locked-open mode (i.e. lemmings could enter without activating the trap). Then, traps were activated with a piece of apple (2004-2015) or with both a piece of apple and a small grape-sized ball of peanut butter mixed with oat and flour (2016-2019). Each lemming trapped was identified to species, sex, weighed, and marked with a PIT-tag or an ear-tag. Ear-tags were used to reduce costs but were employed only during the last primary period of 2016-2019 to avoid false mortalities or emigration due to loss of tags. Inter-annual recaptures are extremely rare due to most lemmings living less than a year (Fauteux et al. 2018b). Recaptured lemmings were once again weighed, their reproductive condition was noted, and the tag number was noted. We assigned individuals to age classes (juveniles or adults) based on their body mass, with adult female lemmings being ≥28 g and adult males being ≥30 g (Fauteux et al. 2015). Traps were then left continuously in the locked-open mode between primary periods. Number of individual lemmings trapped over the years are presented in Table S1.

**Table S1.** Number of individual brown lemmings captured per year for each sex and age group. Lemmings from all primary periods and trapping grids have been pooled to simplify the table.

| Year | Adult females | Adult males | Juvenile females | Juvenile males |
| --- | --- | --- | --- | --- |
| 2004 | 19 | 47 | 11 | 28 |
| 2005 | 13 | 8 | 2 | 3 |
| 2006 | 5 | 5 | 1 | 13 |
| 2007 | 2 | 10 | 2 | 8 |
| 2008 | 56 | 60 | 25 | 57 |
| 2009 | 4 | 7 | 2 | 3 |
| 2010 | 83 | 114 | 19 | 49 |
| 2011 | 169 | 142 | 38 | 61 |
| 2012 | 1 | 9 | 4 | 1 |
| 2013* | 0 | 0 | 0 | 0 |
| 2014 | 181 | 186 | 47 | 62 |
| 2015 | 98 | 101 | 57 | 62 |
| 2016 | 48 | 66 | 26 | 22 |
| 2017 | 3 | 12 | 5 | 4 |
| 2018 | 1 | 1 | 0 | 0 |
| 2019 | 43 | 53 | 19 | 40 |

*No brown lemming was captured in 2013 even though the exact same trapping protocol as the other years was applied.

**Table S2**. Model selection for the survival (φ) analysis of brown lemmings on Bylot Island conducted with E-SURGE. The most parsimonious model and the next two best models are presented. Detection parameter (*p*) was constant for all the most parsimonious models.

| φ | *p* | *K* | Deviance | ΔAICc |
| --- | --- | --- | --- | --- |
| **year**t*+age*sex**t*+grid** | **sex** | **52** | **1943.94** | **0.00** |
| year**t*+age*sex**t*+grid | age+sex | 53 | 1943.87 | 2.01 |
| year**t*+age*sex**t*+grid | age*sex | 54 | 1943.92 | 4.14 |
| year**t*+age*sex**t*+grid | null | 51 | 1951.02 | 5.01 |
| year**t*+age*sex**t*+grid | grid | 54 | 1946.98 | 7.20 |
| year**t*+age*sex**t*+grid | age | 52 | 1950.98 | 7.05 |
| year+age*sex**t*+grid | null | 36 | 1987.87 | 10.93 |
| year**t*+age*sex**t*+grid | year | 64 | 1935.63 | 16.69 |
| year**t*+sex+grid | sex | 41 | 1987.21 | 20.55 |
| age*sex**t*+grid | null | 23 | 2025.02 | 21.53 |
| year**t*+sex**t*+grid | sex | 43 | 1984.43 | 21.89 |
| year**t*+age*sex+grid | null | 40 | 1994.28 | 25.55 |
| year+age*sex+grid+t | null | 25 | 2031.81 | 32.40 |
| year**t*+sex+grid | null | 38 | 2005.94 | 33.10 |
| year**t*+age*sex**t*+grid | year*sex | 77 | 1929.99 | 38.37 |
| year+age*sex+grid | null | 22 | 2044.90 | 39.38 |
| age*sex+grid+*t* | null | 12 | 2067.29 | 41.53 |
| age*sex+grid | null | 8 | 2077.20 | 43.39 |
| year**t**age*sex+grid | null | 105 | 1883.35 | 51.43 |
| year**t*+grid | null | 37 | 2025.45 | 50.56 |
| age*sex+grid**t* | null | 18 | 2063.65 | 50.02 |
| age*sex*grid | null | 17 | 2067.40 | 51.74 |
| age*sex*grid**t* | null | 53 | 1997.51 | 55.65 |
| year+grid | null | 19 | 2074.74 | 63.14 |
| year*t+age*sex | null | 37 | 2043.17 | 68.28 |
| grid | null | 5 | 2107.47 | 67.63 |
| age*sex**t* | null | 20 | 2081.11 | 71.53 |
| grid**t* | null | 15 | 2094.40 | 74.69 |
| year+age*sex | null | 19 | 2093.85 | 82.25 |
| year**t**age*sex | null | 102 | 1927.18 | 88.81 |
| year.*t* | null | 34 | 2080.54 | 99.50 |
| age*sex | null | 5 | 2139.21 | 99.36 |
| year | null | 16 | 2130.06 | 112.39 |
| null | year | 16 | 2135.26 | 117.58 |
| null | age*sex | 5 | 2159.50 | 119.65 |
| null | age+sex | 4 | 2164.78 | 122.93 |
| null | sex | 3 | 2171.28 | 127.42 |
| null | *t* | 6 | 2165.90 | 128.06 |
| null | null | 2 | 2177.52 | 131.66 |
| null | age | 3 | 2175.72 | 131.86 |

year: annual variation; *t*: monthly variation (between primary periods within year); age: categorical variable with two values (adult, juvenile); sex: categorical variable with two values (male, female); grid: categorical variable with four values (wet tundra grid, mesic tundra grid 1, mesic tundra grid 2, predator exclosure).

**Table S3.** Ranking of negative binomial models testing the relationship between the maximum and average distances between the initial capture and recaptures based and population density. The model selected for the results presented in the manuscript is in bold. All models included an offset based on the log-transformed number of recaptures. For each model, the number of parameter (*K*) and the log-likelihood (*LL*) are also shown. The dispersion parameter varied between 0.42 and 0.47 among all models.

| Movements | Model | *K* | Log-likelihood | ΔAICc |
| --- | --- | --- | --- | --- |
| Maximum distance | **density+sex*age** | **6** | **-5773.70** | **0.00** |
|  | density*age+sex | 6 | -5774.79 | 2.19 |
|  | density+sex+age | 5 | -5775.97 | 2.51 |
|  | density*sex*age | 9 | -5772.68 | 4.05 |
|  | density*sex+age | 6 | -5775.97 | 4.53 |
|  | density+sex*age+grid | 9 | -5772.93 | 4.54 |
|  | density*age+sex+grid | 9 | -5773.99 | 6.66 |
|  | density+sex+age+grid | 8 | -5775.23 | 7.12 |
|  | density*sex+age+grid | 9 | -5775.23 | 9.13 |
|  | sex*age | 5 | -5782.11 | 14.80 |
|  | sex*age+grid | 8 | -5780.16 | 16.96 |
|  | density*sex+age+grid | 4 | -5784.55 | 17.65 |
|  | sex+age+grid | 7 | -5782.93 | 20.49 |
|  | age | 3 | -5796.39 | 39.34 |
|  | age+grid | 6 | -5793.61 | 39.82 |
|  | sex | 3 | -5797.10 | 40.74 |
|  | sex+grid | 6 | -5795.08 | 42.75 |
|  | grid | 5 | -5805.22 | 61.01 |
|  | null | 2 | -5808.61 | 61.76 |
| Average distance | **density+sex*age** | **6** | **-5453.47** | **0.00** |
|  | density*sex*age | 9 | -5452.19 | 3.53 |
|  | density+sex*age+grid | 9 | -5452.22 | 3.58 |
|  | density*age+sex | 6 | -5455.60 | 4.27 |
|  | density+sex+age | 5 | -5456.84 | 4.72 |
|  | density*sex+age | 6 | -5456.80 | 6.68 |
|  | density*age+sex+grid | 9 | -5454.43 | 8.00 |
|  | density+sex+age+grid | 8 | -5455.73 | 8.58 |
|  | density*sex+age+grid | 9 | -5455.68 | 10.50 |
|  | sex*age | 5 | -5459.95 | 10.94 |
|  | sex*age+grid | 8 | -5457.38 | 11.88 |
|  | sex+age | 4 | -5463.72 | 16.48 |
|  | sex+age+grid | 7 | -5461.64 | 18.38 |
|  | sex | 3 | -5472.85 | 32.72 |
|  | sex+grid | 6 | -5470.31 | 33.70 |
|  | age+grid | 6 | -5471.00 | 35.07 |
|  | density+sex*age | 3 | -5474.05 | 35.13 |
|  | grid | 5 | -5479.62 | 50.28 |
|  | null | 2 | -5483.39 | 51.78 |

**Table S4.** Ranking of logistic mixed-effects models testing the relationship between the proportions of adult females in reproductive condition (i.e. perforate vagina, lactating or pregnant) and population density. The selected model is in bold.

| Model | *K* | Log-likelihood | ΔAICc |
| --- | --- | --- | --- |
| **density+period+grid** | **8** | **-439.49** | **0.00** |
| period+grid | 7 | -441.57 | 2.13 |
| density+grid | 6 | -444.55 | 6.05 |
| grid | 5 | -446.33 | 7.58 |
| period | 4 | -479.95 | 72.80 |
| density+period | 5 | -479.02 | 72.96 |
| null | 2 | -483.20 | 75.28 |
| density | 3 | -482.39 | 75.67 |

**Table S5**. Model coefficients (*β*) and 95% confidence intervals (CI) for the analysis of the relationship between the proportion of reproductive adult females and population density for each lemming group. Significant relationships are in bold.

| Covariate | *β* | 95% CI |
| --- | --- | --- |
| density | **-0.087** | **[-0.170, -0.004]** |
| periodPP2 | **0.690** | **[0.243, 1.154]** |
| periodPP3 | 0.282 | [-0.190, 0.760] |
| grid LG2 | **-0.820** | **[-1.275, -0.401]** |
| grid LX1 | **-0.952** | **[-1.572, -0.396]** |
| grid Exclosure | **1.448** | **[0.832, 2.130]** |

**Table S6.** Ranking of models testing the relationship between daily change in body mass and ontogenical, seasonal (period), and density-dependent factors based on the Akaike’s information criterion. The selected model is in bold.

| Model | *K* | Log-likelihood | ΔAICc |
| --- | --- | --- | --- |
| initial mass*sex*period+grid | 13 | 72.87 | 0.00 |
| **initial mass*period+grid** | **9** | **68.25** | **0.86** |
| initial.mass*sex*period+density+grid | 14 | 72.89 | 2.07 |
| init.mass*period+sex+grid | 10 | 68.35 | 2.75 |
| initial.mass*period*grid | 18 | 76.12 | 4.16 |
| initial mass*sex+initial mass*period+grid | 11 | 68.36 | 4.83 |
| init.mass*period*grid+sex | 19 | 76.27 | 6.01 |
| Init.mass*period+sex*density+grid | 12 | 68.47 | 6.70 |
| Init.mass*period*density+sex+grid | 14 | 70.06 | 7.74 |
| init.mass*sex+init.mass*period+init.mass* density+grid | 13 | 68.68 | 8.39 |
| init.mass*sex*density*period+grid | 21 | 76.98 | 8.92 |
| init.mass*sex*period | 10 | 64.21 | 11.04 |
| init.mass*period | 6 | 59.48 | 12.23 |
| init.mass*period*density | 10 | 60.44 | 18.57 |
| init.mass+period+grid | 8 | 55.48 | 24.35 |
| init.mass+sex+period+grid | 9 | 55.75 | 25.88 |
| init.mass+sex+period+density+grid | 10 | 55.78 | 27.90 |
| init.mass*sex+period+grid | 10 | 55.75 | 27.95 |
| init.mass*sex*density +period+grid | 14 | 57.16 | 33.54 |
| init.mass+sex*period | 7 | 49.24 | 34.75 |
| init.mass+sex*period*density | 11 | 49.72 | 42.09 |
| init.mass+sex+grid | 8 | -56.27 | 247.83 |
| init.mass+grid | 7 | -57.52 | 248.28 |
| init.mass*sex+grid | 9 | -56.02 | 249.42 |
| init.mass+sex+density +grid | 9 | -56.16 | 249.69 |
| init.mass+density +grid | 8 | -57.27 | 249.84 |
| init.mass+sex | 5 | -60.91 | 250.96 |
| init.mass | 4 | -62.32 | 251.74 |
| init.mass*density+grid | 9 | -57.26 | 251.90 |
| init.mass*sex | 6 | -60.74 | 252.67 |
| init.mass+density | 5 | -62.02 | 253.17 |
| init.mass*sex+init.mass*density+grid | 11 | -55.93 | 253.39 |
| init.mass*sex*density+grid | 13 | -54.57 | 254.88 |
| init.mass*sex*grid | 18 | -52.19 | 260.77 |
| sex | 4 | -143.86 | 414.81 |
| density | 4 | -154.94 | 436.98 |
| grid | 6 | -154.60 | 440.39 |
| Null | 3 | -157.69 | 440.45 |

**
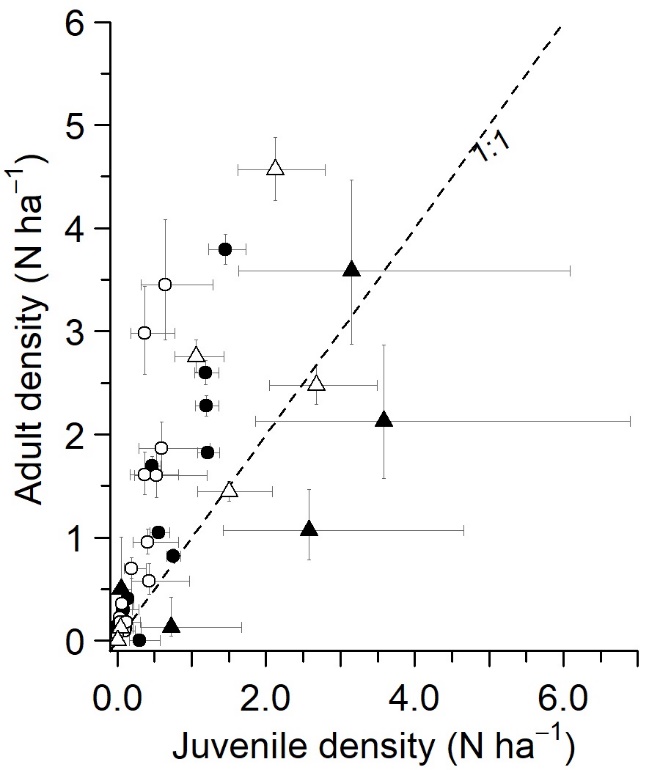
**

**Figure S1.** Relationship between adult (adult females, ≥28 g; adult males ≥30 g) and juvenile density at Bylot Island, 2004-2019. Black circles: wet meadow trapping grid 1; white circles: mesic trapping grid 1; black triangles: mesic trapping grid 2; white triangles: predator exclosure trapping grid. The dashed line is a visual guideline and represents a 1:1 ratio.


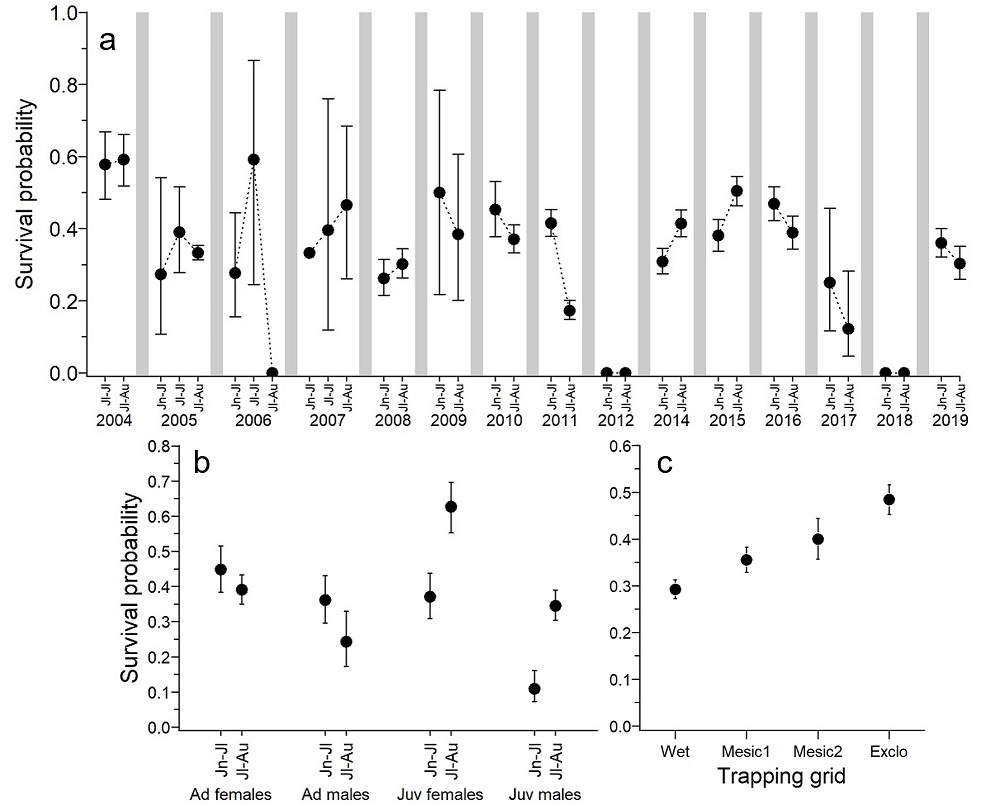


**Figure S2.** Survival estimates of brown lemmings at Bylot Island from the most parsimonious model (see Table S2). Average survival estimates with their respective 95% confidence intervals are presented for each trapping period and year (a), lemming groups based on sex and age (juveniles vs adults) (b), and trapping grid (c). Grey bars indicate winters. Jn-Jl: mid-June to early July for 2004-2007 in a, mid-June to mid-July elsewhere; Jl-Jl: early July to late July; Jl-Au: late-July to mid-August for 2004-2007 in a, mid-July to mid-August elsewhere; Wet: wet trapping grid; Mesic1: mesic grid 1; Mesic2: mesic grid 2; Exclo: predator exclosure grid. No survival probabilities are reported for 2013 because no lemming was captured that year.
